# The cost-utility of catheter ablation of atrial fibrillation: a systematic review and critical appraisal of economic evaluations

**DOI:** 10.1186/1471-2261-13-78

**Published:** 2013-09-26

**Authors:** Mattias Neyt, Hans Van Brabandt, Carl Devos

**Affiliations:** 1 Belgian Health Care Knowledge Centre (KCE), Doorbuilding Kruidtuinlaan 55, B-1000, Brussels, Belgium

**Keywords:** Atrial fibrillation, Catheter ablation, Cost-Benefit analysis, Review

## Abstract

**Background:**

A health technology assessment (HTA) of catheter ablation for atrial fibrillation (CA-AF) was commissioned by the Belgian government and performed by the Belgian Health Care Knowledge Centre (KCE). In this context, a systematic review of the economic literature was performed to assess the procedure’s value for money.

**Methods:**

A systematic search for economic literature about the cost-effectiveness of CA-AF was performed by consulting various databases: CRD (Centre for Reviews and Dissemination) HTA and CDSR (Cochrane Database of Systematic Reviews) Technology Assessment, websites of HTA institutes, NHS EED (NHS Economic Evaluation Database), Medline (OVID), EMBASE and EconLit. No time or language restrictions were imposed and pre-defined selection criteria were used. The two-step selection procedure was performed by two persons. References of the selected studies were checked for additional relevant citations.

**Results:**

Out of 697 references, seven relevant studies were selected. Based on current evidence and economic considerations, the rationale to support catheter ablation as first-line treatment was lacking.

The economic evaluations for second-line catheter ablation included several assumptions that make the results rather optimistic or subject to large uncertainty. First, overall AAD (antiarrhythmic drugs) use after ablation was higher in reality than assumed in the economic evaluations, which had its impact on costs and effects. Second, several models focused on the impact of ablation on preventing stroke. This was questionable because there was no direct hard evidence from RCTs to support this assumption. An indirect impact through stroke on mortality should also be regarded with caution. Furthermore, all models included an impact on quality of life (QoL)/utility and assumed a long-term impact. Unfortunately, none of the RCTs measured QoL with a generic utility instrument and information on the long-term impact on both mortality and QoL was lacking.

**Conclusions:**

Catheter ablation is associated with high initial costs and may lead to life-threatening complications. Its cost-effectiveness depends on the belief one places on the impact on utility and/or preventing stroke, and the duration of these effects. Having no hard evidence for these important variables is rather troublesome. Although the technique is widely spread, the scientific evidence is insufficient for drawing conclusions about the intervention’s cost-effectiveness.

## Background

Atrial fibrillation (AF) is a common cardiac arrhythmia characterised by an irregular and fast heartbeat. Its prevalence increases with age, and up to 15% of over-eighty-year-olds have been diagnosed with AF [1]. AF may cause symptoms of palpitations, shortness of breath and fatigue. It can also lead to heart failure and stroke. Treatment is primarily aimed at restoring and maintaining sinus rhythm and preventing stroke. Rhythm control can be achieved by means of electric cardioversion or by using antiarrhythmic drugs (AAD) such as flecainide, propafenone or amiodarone. The risk of stroke can be considerably reduced by anticoagulants. Unfortunately, in many patients antiarrhythmic drugs are not effective or poorly tolerated [2]. Then, treatment is limited to rate control, i.e. slowing the heart rate that is often inappropriately fast in AF. This can be achieved by means of beta-blockers and some calcium channel blockers. Of note, a clinical advantage of rhythm control over rate control could never be documented in clinical trials [3,4]. Selected and severely symptomatic patients that do not adequately respond to drugs can be offered an invasive treatment known as catheter ablation (CA). It involves the introduction of a catheter into the heart to selectively destroy specific areas in the left atrium that contain cells that trigger the onset of AF.

In randomised controlled trials (RCTs) that enrolled selected patients, it has been demonstrated that in the short term, CA can be more effective than antiarrhythmic drugs for symptom control [5]. The long-term effect of CA however is not known. Moreover, an effect on hard endpoints such as stroke or mortality could not be demonstrated so far.

Several authors have claimed CA for AF being a cost-effective intervention without performing a critical assessment of the underlying studies supporting this statement. In the context of a health technology assessment (HTA) of CA that was commissioned by the Belgian government, we performed a systematic review of the literature on the cost-effectiveness of this procedure. The conclusions and recommendations of this HTA report provide advice to our policy makers in order to take evidence-based reimbursement decisions. The results presented in this paper are extracted from this report.

## Methods

In August 2012, a systematic search for economic literature about the cost-effectiveness of CA-AF was performed by consulting various databases. First of all, reviews on this topic were searched by consulting the CRD (Centre for Reviews and Dissemination) HTA and CDSR (Cochrane Database of Systematic Reviews) Technology Assessment databases. The websites of HTA institutes mentioned on the INAHTA (International Network of Agencies for Health Technology Assessment) website (http://www.inahta.org) were also consulted. Websites of non-member HTA institutes such as NICE (http://www.nice.org.uk) were also checked for relevant analyses. Searching for relevant reports on these websites was performed independently by two persons (MN and HVB). Furthermore, The NHS EED (NHS Economic Evaluation Database), Medline (OVID), EMBASE and EconLit databases were searched to retrieve both full economic evaluations and reviews of full economic evaluations of CA-AF. No restrictions on the time period and language were imposed. Details of this search strategy are available in the appendix of the full HTA report [5]. The search strategy in these databases was performed by one researcher, transparently reported, and validated afterwards.

All retrieved references were assessed against pre-defined selection criteria (Table 1). The population was not restricted to a specific type of atrial fibrillation. Both paroxysmal, persistent and permanent AF populations were eligible. It was expected that the retrieved economic evaluations focused on radiofrequency CA since the evidence was mainly restricted to this type of intervention. The comparator was a rate or rhythm control strategy. The design was restricted to full economic evaluations, i.e. studies comparing at least two alternative treatments in terms of costs and outcomes. Both trial-based and modelled economic evaluation studies were eligible for inclusion in this systematic review.

**Table 1:** Economic evaluation selection criteria

	**Inclusion criteria**	**Exclusion criteria**
Population	Patients with atrial fibrillation	Other populations
Intervention	(Radiofrequency) catheter ablation	Other interventions
Comparator	Rate or rhythm control, including electric cardioversion	Other types of catheter ablation or surgical procedures
Design	Full economic evaluations	Other designs such as cost calculations

**Figure 1:**
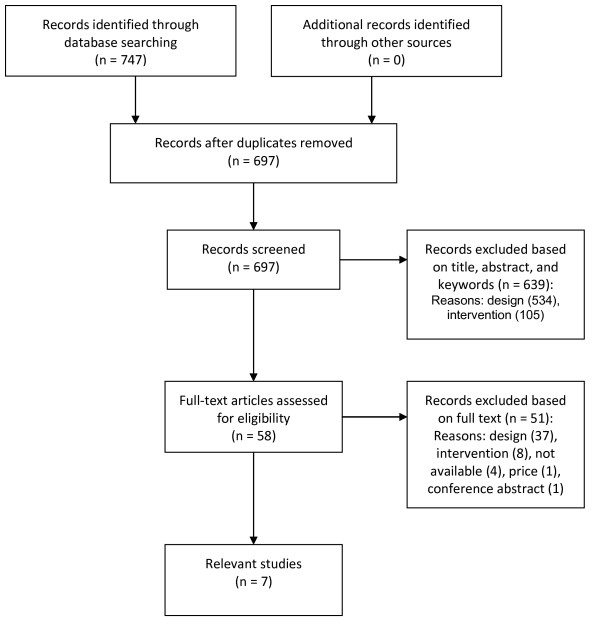
Selection of relevant articles.

The selection of relevant articles was performed in a two-step procedure: initial assessment of the title, abstract, and keywords, followed by a full-text assessment of the selected references. This procedure was performed by two persons, one health economist and a cardiologist, both experienced in performing systematic reviews and HTA. Reference lists of the selected studies were checked for additional relevant citations. Figure 1 provides the flow chart of this process. Most articles were excluded because of not being a full economic evaluation (design) or including an inappropriate in tervention. In the end, seven relevant studies were selected [6-12]. Two studies represented the same analysis and are discussed as one study [9,12]. These full economic evaluations were summarized by a health economist in an in-house structured data extraction sheet. These working documents provided the basis to make this overview in which the models’ input variables were compared with the systematically identified evidence and Belgian real-world practice data. This critical appraisal was performed by the research team.

The clinical evidence necessary for the critical appraisal of the input variables in the economic evaluations was also gathered in a systematic way as part of the full HTA report [5]. The medical research question focused on the effect of catheter ablation in comparison to medical therapy, for patients suffering from atrial fibrillation, on heart rhythm, symptoms and quality of life, the occurrence of complications and on survival. Details of the search strategy are available in the full HTA report [5]. Nine randomised controlled trials (RCTs), published from 2003 to 2011 were identified and summarized [5]. Two recent systematic reviews [13,14] and three recent HTA reports (Canada, US, Sweden) [6,10,15] were also identified and included the same major RCTs from our systematic search. In addition, we had Belgian administrative data at our disposal which allowed us to retrieve real-world cost data and drug administration after catheter ablation. Details of the Belgian practice are published elsewhere [16].

## Results

In the following paragraphs, we provide an overview of all input variables, results and conclusions from the published economic evaluations. Initially, the data are provided as published in the economic evaluations. Full details are available in an Additional file (Additional file 1: Table S1–Table S10). This information will then be critically appraised in our discussion.

### General information

All studies were published between 2006 and 2010. They were performed for Canada, [6] the US, [7,10,11] Sweden [8] and the UK [9,12]. All studies performed a cost-utility analysis (CUA) based on a Markov model with in some cases a decision tree modelling the events during the first year. The time horizon was 5 years or lifetime. The discount rate for costs and health outcomes reflected national guidelines (3%, [7,8,10,11] 3.5% [9,12] or 5% [6] for both costs and outcomes). The perspective was a payer or societal perspective. However, in the latter case, productivity and travel costs were not included and the analyses rather reflected the payer perspective (Additional file 1: Table S1).

### Population

The populations were different across studies. Most of the studies included (mainly) male patients with paroxysmal AF who were unsuccessfully treated with an AAD. However, some analysis also modelled first-line CA [7,10] or included persistent AF patients [8,10]. Some studies explicitly mentioned to include (moderately to highly) symptomatic patients [8,10]. Age and stroke risks also differed across studies (Additional file 1: Table S1).

### Intervention and comparator

The assessed intervention was radiofrequency catheter ablation for treatment of atrial fibrillation. In all cases, AAD was the comparator. The study of Chan et al. [7] assessing first-line CA also included both rate control and rhythm control with AAD as a comparator (Additional file 1: Table S1).

### Catheter ablation and procedural risks

The cost for the initial procedure ranged between about $10,000 and $16,500 per procedure. The average number of procedures was about 1.3 (range: 1.25–1.4). The most often included adverse events were procedural death (0.05–0.1%), stroke (0.3–0.8%), transient ischemic attack (TIA) (0.2–0.4%), cardiac tamponade (0.7–1.22%), and pulmonary vein (PV) stenosis (0.4–1.6%). Some models included a general cost for procedure complications [7,8,10], while other models made a distinction between different types of complications (see Additional file 1: Table S2) [6,9,11,12].

### AADs, rate control and anticoagulation

Table S3 (Additional file 1) presents the costs for AAD, rate control and anticoagulation. The differences reflect not only price differences of drugs, but also differences in the elements included in the cost estimate (e.g. whether or not hospitalisations are included in the AAD cost estimate). Two studies mentioned costs for rate control [7,10], which are lower than those for AAD treatment. Assumptions relating to the use of anticoagulation treatment was somewhat different across studies: Assasi et al. [6] assumed that AF ablation patients discontinued warfarin three months after their procedure; Chan et al. [7] assumed patients with restored sinus rhythm continued warfarin therapy for six more months before transitioning to the use of aspirin; the other studies mentioned anticoagulation depended on the stroke risk, [8] or would continue as appropriate regardless of whether AF had recurred, [10] with equivalent practices in all treatment groups [11].

### Stroke, bleeding and drug toxicity

Table S4 (Additional file 1) provides an overview of stroke costs and other costs such as those for bleeding or drug toxicity. The probabilities of these events are shown in Table S5 and Table S6 (Additional file 1). Half of the models assumed that the annual stroke risk was lower with a normal sinus rhythm [6,7,9,12]. The other half did not assume a reduction in stroke events in their base case analysis [8,10,11]. The bleed and toxicity risks also differed in most studies depending on the drugs taken (Additional file 1: Table S5 and Table S6). Differences in mortality risk between the intervention and control arm was modelled indirectly, e.g. through the different stroke risks (Additional file 1: Table S6). Only Reynolds et al. [11] explicitly mentioned the projected all-cause mortality was equivalent between both groups.

### Normal sinus rhythm

Table S7 (Additional file 1) provides an overview of the modelled efficacy in both the ablation arm and for the comparators. The model inputs of first-line treatments [7,10] were incomparable with those after unsuccessful AAD treatment. A normal sinus rhythm was achieved in 74% [9,12] up to 90% [11] with catheter ablation. For non-first line AAD treatment this was between 9% [8] and 37% [9,12] at one year. In the models including first-line treatments, [7,10] patients under rate control were assumed to convert spontaneously to a normal sinus rhythm in 38% of cases. The annual probability of AF recurrence after the first year was not always explicitly mentioned. In the 2nd-line treatment models of Assasi et al. [6] and Rodgers et al., [9,12] it was lower than 4% in the ablation arm.

### Utilities

Utility values were modelled indirectly through several assumptions. Most models started from age- and gender-specific general population utility values and took into account a (dis)utility for certain health states (e.g. normal sinus rhythm or AF) and events (e.g. stroke or procedure complications) [6,8-12]. In contrast to all other models, Chan et al. [7] assigned a utility value of 1, i.e. perfect health, to patients in normal sinus rhythm. We refer to Table S8 (Additional file 1) for details on the assumed (dis)utility for health states and events.

### Results of the identified economic evaluations

#### Base case

Table S9 (Additional file 1) presents the results of the base case analyses. For second-line catheter ablation, the base case average incremental cost-effectiveness ratios (ICERs) ranged from less than £8 000 per QALY for different CHADS2 scores, [9,12] up to about $60 000 for a CHADS2-score of 2 in paroxysmal AF patients [6]. In patients with persistent AF, the results were less favourable [10].

Two studies modelled first-line catheter ablation. One indicated the needed relative reduction in stroke risk to reach a pre-specified ICER threshold, referring to the hypothetical character of the analysis [7]. Similarly as for second-line catheter ablation, Ollendorf et al. [10] found less favourable results for first-line catheter ablation in persistent AF patients in comparison to paroxysmal AF.

#### Sensitivity analyses

A selection of the most important results from the various sensitivity analyses is presented in Table S9 (Additional file 1). The most determining variables were: the difference in utility between the intervention and comparator group, the applied time horizon, and the impact on preventing stroke. Shorter time horizons and/or smaller utility differences easily increased the average ICERs to more than $100 000 per QALY [6,11]. The analysis of Rodgers and McKenna [9,12] indicated stroke risk didn’t have much influence on results, while other models provided better results for high-risk patients [6-8]. This was of course dependent on the initial modelling assumptions and the baseline stroke risk.

#### Authors’ conclusions

Table 2 and Table S10 (Additional file 1) provide an overview of the conclusions of all identified studies. For first-line catheter ablation, the conclusions reflected the hypothetical character of the analyses and the lack of evidence: “if sufficiently high CA efficacy rates in restoring sinus rhythm translate into lower morbidity…”, [7] and “unproven with potential” efficacy for catheter ablation of patients with paroxysmal AF or long-standing persistent AF and heart failure [10].

**Table 2:** Conclusions of retrieved economic evaluations on CA’s cost effectiveness

**First-line catheter ablation**	
Chan et al., 2006 (US) [7]	In patients with AF, catheter ablation is unlikely to be cost-effective in patients at low risk for stroke. In moderate-risk patients, catheter ablation may be cost-effective if sufficiently high efficacy rates in restoring sinus rhythm translate into lower morbidity.
**First- and second-line catheter ablation**	
Ollendorf et al., 2010 (US) [10]	No explicit conclusion on the intervention’s cost effectiveness is drawn.
	There is only a high certainty of a small benefit for second-line ablation in paroxysmal AF patients. In other populations and for first-line ablation there is a potential but unproven benefit.
**Second-line catheter ablation**	
Assasi et al., 2010 (Canada) [6]	The primary economic evaluation using a five-year time horizon found the incremental cost per QALY of AF ablation compared with AAD to be $59 194.
Eckard et al., 2009 (Sweden) [8]	The radiofrequency ablation treatment strategy was associated with reduced cost and an incremental gain in QALYs and was considered a cost-effective treatment strategy compared to the AAD in a lifetime perspective.
Reynolds et al., 2010 (US) [11]	Catheter ablation with/without AAD for symptomatic, drug-refractory paroxysmal AF appears to be reasonably cost-effective compared with AAD therapy alone from the perspective of the US health care system. The ICER for catheter ablation versus AAD was $51 431 per QALY applying a 5-year time horizon.
Rodgers et al., 2008 (UK) [9,12]	The overall conclusions regarding the cost-effectiveness of catheter ablation appear to require that the QoL benefits are maintained for more than 5 years and/or that normal sinus rhythm has prognostic value in preventing the risk of stroke. If neither of these is considered to be realistic then the cost-effectiveness of catheter ablation remains highly uncertain.
McKenna et al., 2009 (UK) [9,12]	

For second-line catheter ablation, some authors were very confirmative in considering CA a cost-effective intervention [8,11]. Others referred to the uncertainty surrounding decisive variables: e.g. “it requires that the quality of life (QoL) benefits are maintained for more than 5 years and/or that normal sinus rhythm has prognostic value in preventing the risk of stroke” [9,12]. The report of Ollendorf et al. [10] mentioned there was a high certainty of a small benefit only for secondary catheter ablation in paroxysmal patients. However, no explicit conclusion on the intervention’s cost-utility was stated.

In our discussion, based on current evidence, we will show why the modelled results are probably overoptimistic.

## Discussion

In this discussion, several input variables are critically appraised by linking them to current evidence, gathered in a Belgian HTA report performed by the Belgian Health Care Knowledge Centre (KCE) in which a systematic review and critical appraisal of the clinical evidence was performed and in which the Belgian real-world practice is analysed based on administrative data (see full HTA report [5] for details).

### Rate control

Most of the economic models include patients with paroxysmal AF unsuccessfully treated with AAD. Even though evidence on the effectiveness of first-line ablation to restore sinus rhythm or have an impact on patient-relevant outcomes is lacking, two studies also model the cost-effectiveness of first-line ablation. In the study of Chan et al. [7] amiodarone was both less effective and more costly, and thus dominated by rate control therapy (Additional file 1: Table S9). Ollendorf et al. [10] concluded efficacy of primary catheter ablation is unproven but has potential (Additional file 1: Table S10).

In an economic evaluation based on the AFFIRM study, “*a mean survival gain of 0.08 year (P = 0.10) was observed for rate control in comparison with antiarrhythmic drugs (rhythm control). Patients in the rate-control group used fewer resources (hospital days, pacemaker procedures, cardioversions, and short-stay and emergency department visits). Rate control costs $5 077 less per person than rhythm control*” [17]. The authors of this study concluded that “*Rate control is a cost-effective approach to the management of atrial fibrillation compared with maintenance of sinus rhythm with AAD in patients with atrial fibrillation similar to those enrolled in AFFIRM*” [17]. Reynolds et al. [11] assumed that patients were seeking rhythm control strategies because of dissatisfaction with rate control alone. In economic evaluations, including the previously most cost-effective alternative as a comparator, i.e. working on the efficiency frontier, is of major importance since this might have a big influence on cost-effectiveness results, conclusions and recommendations [18]. Therefore, taking into account the results of the AFFIRM study, rate control should also be considered as a relevant comparator in economic evaluations of catheter ablation.

The use of catheter ablation in first line should clearly be distinguished from second-line use. Based on current evidence [5] and economic considerations, the rational to support catheter ablation as first-line treatment is lacking and both rate/rhythm control should be considered first. However, based on real-world Belgian data of 830 patients, 84.2% of patients were treated with amiodarone or sotalol, which both have rate and rhythm control properties, or a combination of an antiarrhythmic drug and a rate control drug before they underwent their first ablation, indicating that up to 15.8% of patients underwent catheter ablation as a first line therapy of AF [5,16].

### Procedural complications

The bulk of published data on AF ablation comes from selected centres of excellence [11]. Data on complications from these centres may underestimate real-world complication rates. Data from real-world registers or surveys may provide more realistic values. Some studies [9,11,12] refer to the worldwide survey conducted by Cappato and colleagues [19]. Not all complications mentioned in this survey are included in the models. Whereas complications are estimated to be around 5%, [5] all but one [10] of the models include only 3% or less complications. This difference might be explained by the fact that some of these complications occur during the initial hospitalisation, and therefore, related costs are already included in those of the ablation procedure. If this is not the case, the underestimation of costs will favour the ablation group. Furthermore, the survey of Cappato et al. [19] only had a 23% response rate. As mentioned by Rodgers, [12] the findings of this survey have a clear potential for bias, most likely in favour of ablation, i.e. by overestimating success rates and/or underestimating complications. On the other hand, some experts remark that increased experience and/or concentration of the procedure in the most experienced centres may lead to lower rates of complications than those reported by Cappato and colleagues.

### Use of drugs

The economic evaluations make several assumptions towards the use of drugs. One study [6] assumed that AF ablation patients discontinue warfarin three months after their procedure. This results in different costs and bleeding risks between AF ablation patients and AAD-treated patients. However, this does not reflect reality and favours the ablation group. Reynolds et al. [11] assume that ablation patients would not be treated with amiodarone after failed ablation. Belgian real-world data show this is not the case. Between 3 and 12 months after the procedure, i.e. with a 3-months blanking period, 54.9% of patients were using at least temporarily an AAD. Between 3 and 24 months, this was 60.7% [5,16]. In fact, these data show that overall AAD drug use after ablation is higher in Belgium than in the economic evaluations. Whatever the reason might be (e.g. failure of the CA-AF procedure or inappropriate use), it has an impact on both costs and effects. First, costs will be higher in the ablation group. Second, the modelled adverse events linked to these drugs (e.g. bleeding and pulmonary toxicity) are thus underestimated in the ablation group. This leads to overoptimistic results for catheter ablation in the economic evaluations.

The HTA was performed to provide recommendations to the Belgian policy makers. Therefore, the Belgian situation was considered very relevant and Belgian real-world data on drug use after catheter ablation were analysed. However, it is not clear in how far the utilisation and clinical management in Belgium is applicable to other countries and healthcare systems.

### Stroke and mortality

Several models focus on the impact of ablation on preventing stroke [6,7,9,12]. However, there is no direct hard evidence from RCTs to support this assumption [5]. Unless evidence from ablation therapy on stroke is provided in a well-performed RCT with optimal treatment in the control group, results of models that assume a major impact on preventing stroke remain questionable.

The evidence does not suggest that ablation is associated with increased mortality [12]. However, the opposite is also true. Nevertheless, an impact on mortality is modelled through assuming a different stroke risk and including an immediate stroke mortality and an increased mortality risk afterwards (Additional file 1: Table S6). If the impact on preventing stroke is not supported by hard evidence, then an indirect impact through preventing stroke on mortality should also be regarded with caution.

All models mention to perform a cost-utility analysis. However, with the exception of one study, no results are presented in life-years gained. Therefore it is not possible to separately assess the modelled impact of mortality and QoL on results. Only one study provided such information. Through their base case assumption that ablation had a small procedure-related stroke risk and did not impact the long-term stroke risk, ablation was estimated to be more expensive and provide less life-years than rhythm control [10]. Reynolds [11] also mentioned the projected all-cause mortality was equivalent between groups (7.7% ablation versus 7.8% AAD) (Additional file 1: Table S6). It is not clear whether or not other models included a large impact on mortality. If this would be the case, then this would be questionable since no hard evidence is available to support an increased/decreased stroke and/or mortality risk.

### Quality of life

There is evidence from RCTs that ablation improves QoL in the short-term, measured with the generic profile SF-36 instrument. Unfortunately, none of the RCTs measured QoL with a generic utility instrument and information on the long-term impact on QoL is lacking [5]. Notwithstanding, all models include an impact on QoL and assume such a long-term impact. Some assumptions make the results of the economic evaluations rather optimistic or subject to large uncertainty.

With the exception of one study, [10] none of the economic evaluations include a utility loss for the initial ablation procedure (Additional file 1: Table S8). Although this impact might be relatively small, it is applicable to all patients in the ablation group.

Chan et al. [7] apply a utility of 1, i.e. perfect health, for patients well in sinus rhythm, and values close to 1 for healthy patients taking aspirin, warfarin or amiodarone (Additional file 1: Table S8). However, the utility of an average healthy population is not equal to 1, which is shown in the other models that use age- and gender-specific general population values. As a result, the modelled incremental effect is very probably too large in this study.

The RCTs have not measured QoL with a generic utility instrument. Consequently, all models try to rely on best available data to include the incremental impact on quality-adjusted life years. For example, Reynolds et al. [11] derived utilities for 3 separate populations of patients with AF to estimate the likely changes that might be observed after successful ablative or drug therapy. For drug-treated patients, SF-12 data from the FRACTAL registry [20] were transformed to utilities. For ablation patients, SF-36 data from a prospective cohort of patients undergoing catheter ablation at a medical centre were transformed to utilities [11]. And finally, utilities were calculated using SF-36 data for patients enrolled in the A4 trial [21] to estimate the comparative changes in utility for patients treated with drugs versus ablation. However, this study had a 67% rate of crossover to ablation in the AAD group. In general, indirect estimation of utilities, based on different studies, measured with different instruments, and transformed to utilities through mapping is prone to very large uncertainty and should be regarded with caution.

Most studies include utility decrements for specific health states or events. The evidence base for these decrements is most of the time lacking. In the UK study [9,12] a different decrement is included for the same health state after ablation versus AADs, while decrements for adverse events are modelled separately. For example, the decrement for atrial fibrillation is 0.0034 in the ablation group, while this is 0.0925 in the AAD group. This is in favour of the ablation group. The impact on QoL is also modelled through the impact on preventing stroke. Again, it is very important to have hard evidence on this stroke endpoint in order to allow reliable cost-effectiveness calculations.

Disutilities are also modelled for drug related events. However, the real-world Belgian data indicate that a large part of the ablation group still takes one or more of these drugs after the intervention. Not taking this into account underestimates the adverse events and impact on QoL and thus is in favour of the ablation intervention.

It is stated that “*it should be recognised that the QoL estimates applied in the model remain highly uncertain*” [12]. This applies to all identified models. Even so, the results and sensitivity analyses show that the impact on QoL is a determinant factor for the cost-effectiveness of ablation. Therefore, it is desirable to have better data to support these economic evaluations. In future research, QoL should be measured with a generic utility instrument (such as the EQ-5D) in a properly performed RCT.

### Time horizon

Most analyses use a lifetime horizon in their base case analysis (Additional file 1: Table S1). Evidence for longer-term benefits of ablation is lacking. Extrapolating potential benefits reported over shorter time horizons is standard in economic evaluations. Nevertheless, the reliability of results becomes increasingly uncertain, especially in this case of ablation where both short and long-term evidence on the catheter ablation impact on mortality, stroke, and quality of life (utilities) is lacking. Comparing input variables from the models with more recently published data also indicates that extrapolations are probably too optimistic. For example, the annual probability of AF recurrence is less than 4% in several studies (Additional file 1: Table S7) [6,7,9,12]. According to experts, this should be rather between 6% and 9% [22]. In Belgium, a repeat procedure during the second year after the index ablation was performed in about 9% [5]. A 90% success rate (Additional file 1: Table S7) also seems rather optimistic in comparison with data from the medical review. Other assumptions, such as not allowing for repeat ablation procedures after the first 12 months [9,12] are not in accordance with reality. These assumptions are clearly in favour of the ablation arm, especially in models with a longer time horizon.

### Uncertainty

The sensitivity analyses of the economic evaluations show that the most important variables in the models are the catheter ablation’s impact on preventing stroke, the impact on quality of life and whether or not this effect is maintained in the long term. However, as discussed in the previous paragraphs, the assumed impact of catheter ablation on these variables is questionable since no hard evidence is available to support these assumptions and Belgian experience retrieved from real-world administrative data, e.g. on the use of drugs after ablation, is tempering previous expectations.

## Conclusion

There seems to be no good arguments to support a broad first-line use of catheter ablation for the treatment of AF based on currently available evidence and the published economic evaluations. For second-line catheter ablation, the results of the published economic evaluations are diverse. On the one hand, there are those that consider catheter ablation a cost-effective intervention for the treatment of (paroxysmal) AF [8,11]. On the other hand, some authors do not explicitly make a conclusion on the intervention’s cost effectiveness [10] or refer to the uncertainty surrounding decisive variables [9,12]. Our critical appraisal indicates that the most determining input variables for a cost-effectiveness assessment of catheter ablation of AF are the impact on utility and/or preventing stroke, and the duration of these effects. However, it now appears that there are no good utility data, especially not in the long term, and that evidence on stroke impact is lacking. Long-term extrapolation without hard short-term evidence on these endpoints is even more uncertain.

All of the reviewed economic evaluations suffer one or more of the previous discussed shortcomings. Some authors have acknowledged this by remarking that “*the studies are of insufficient size and duration to evaluate the impact on stroke, heart failure, and mortality*” [6] or that there is “*no evidence on long-term outcomes of mortality and stroke*” [10]. This makes the results rather hypothetical. In fact, based on current knowledge, it is difficult to assess whether catheter ablation represents efficient use of limited resources. It is a certainty that catheter ablation is associated with high initial costs and may lead to life-threatening complications. Its cost-effectiveness depends on the belief one places on long-term outcomes. Modelling is hardly ever without assumptions. However, having no hard evidence for the most important variables is rather troublesome. Better evidence in the medium-term (1–5 years) is necessary before extrapolating to very long-term outcomes (>5 years). In the Belgian HTA report, it was decided not to model the cost-effectiveness of catheter ablation. The calculations would be prone to the same uncertainty as all previous models. This is in line with the 2010 SBU (The Swedish Council on Health Technology Assessment) report that concluded that “*The scientific evidence is insufficient for drawing conclusions about the cost-effectiveness of the method since its long-term effects are uncertain*” [15]. We fully agree with that. Since evidence on hard endpoints is lacking, catheter ablation should not be presented to policy makers as a cost-effective intervention. Hypothetical estimations should be distinguished from evaluations based on reliable evidence.

Furthermore, patients should be correctly informed about the adverse events entailed with this invasive procedure, the probabilities for a repeat procedure, the chance that they remain on drugs, etc. To the European bodies, we recommend that they should not only demand that safety and performance data on devices are furnished but also that their clinical effectiveness is demonstrated before these devices become widely used in daily practice. And finally, in further research, RCTs are needed to compare catheter ablation with rate control drugs. Crossovers to catheter ablation in the course of the study should be avoided. Information about patient-relevant endpoints (mortality, quality of life measured with a utility instrument, stroke and other side effects) should be compiled in the course of these in order to be able to make more reliable and robust (economic) evaluations and policy recommendations.

## Competing interests

The current study was performed by researchers of the Belgian Health Care Knowledge Centre, a Belgian governmental agency. No external funding was received for this study. The authors declare that they have no competing interests.

## Authors’ contributions

All authors participated in the concept and design of the study. MN, HVB and CD participated in data collection and data analysis. MN and HVB performed the review of the economic evaluations. All authors read and approved the final manuscript.

## Pre-publication history

The pre-publication history for this paper can be accessed here:

http://www.biomedcentral.com/1471-2261/13/78/prepub

## Supplementary Material

Additional file 1: Table S1 General information on economic evaluations. **Table S2** Information on costs (part 1: CA procedure and complications). **Table S3** Information on costs (part 2: drugs). **Table S4** Information on costs (part 3: stroke and other costs). **Table S5** Risk information (part 1: stroke and bleeding risk). **Table S6** Risk information (part 2: toxicity and mortality risk). **Table S7** Efficacy of intervention and comparator(s). **Table S8** Utilities in the economic evaluations. **Table S9** Results of the economic evaluations. **Table S10** Conclusions of the economic evaluations. Click here for file
