# Supplementary material for: The cost-utility of catheter ablation of atrial fibrillation: a systematic review and critical appraisal of economic evaluations
Source: BMC Cardiovasc Disord. 2013 Sep 26;13:78. doi: 10.1186/1471-2261-13-78 (PMC3849361; doi:10.1186/1471-2261-13-78)
Supplement: Additional file 1: Table S1 — General information on economic evaluations. Table S2 Information on costs (part 1: CA procedure and complications). Table S3 Information on costs (part 2: drugs). Table S4 Information on costs (part 3: stroke and other costs). Table S5 Risk information (part 1: stroke and bleeding risk). Table S6 Risk information (part 2: toxicity and mortality risk). Table S7 Efficacy of intervention and comparator(s). Table S8 Utilities in the economic evaluations. Table S9 Results of the economic evaluations. Table S10 Conclusions of the economic evaluations. [file 1471-2261-13-78-S1.pdf]

Additional file

Table S1: General information on economic evaluations

Reference	Assasi et al., 2010	Chan et al., 2006	Eckard et al., 2009	Ollendorf et al., 2010	Reynolds et al., 2010	Rodgers et al., 2008<br>McKenna et al., 2009
Country	Canada	US	Sweden	US	US	UK
Currency	Canadian dollar (2010)<br><br>(1 CAD = 0,7305 EUR)*	US dollar (2004)<br><br>(1 USD = 0,7702 EUR)	US dollar (2006)<br><br>(1 USD = 0,7702 EUR)	US dollar (2010)<br><br>(1 USD = 0,7702 EUR)	US dollar (2001-2006)<br><br>(1 USD = 0,7702 EUR)	UK pounds sterling (2006)<br>1 GBP = 1,1631 EUR
Conflict of interest	Yes	Not reported	Not reported	Not reported	Yes	Not reported
Population	65-year-old males<br>Paroxysmal AF<br>Unsuccessful AAD<br>CHADS2 score of 2	55- and 65-year-old AF<br>First-line treatment<br>Moderate/low stroke risk	Symptomatic patients<br>Paroxysmal/persistent AF<br>Unsuccessful AAD	Moderately to highly symptomatic atrial fibrillation, first-line or after AAD failure:<br>- 60, male, paroxysmal AF<br>- 65, male, long-standing persistent AF and HF<br>- 75, male, hypertension and diabetes mellitus and persistent AF.	60-year-old males<br>Paroxysmal AF<br>Unsuccessful AAD<br>Without severe structural heart disease	Mean age 52, 80% male<br>AF (majority paroxysmal)<br>Unsuccessful AAD
Intervention	Radiofrequency ablation	Radiofrequency ablation	Radiofrequency ablation	Radiofrequency ablation	Radiofrequency ablation with/without AAD	Radiofrequency ablation without long-term AAD
Comparator	AAD (amiodarone)	AAD (amiodarone) or rate control therapy	AAD	AAD	AAD	AAD (amiodarone)

Type of analysis	CUA	CUA	CUA	CUA	CUA	CUA
Design	One-year decision tree and a longer-term Markov model	Markov model	One-year decision tree and a longer-term Markov model	Markov model	Markov model	One-year decision tree and a longer-term Markov model
Time horizon	5 years	Lifetime	Lifetime	Lifetime	5 years	Lifetime
Discount rate	5% for both costs and health outcomes	3% for both costs and health outcomes	3% for both costs and health outcomes	3% for both costs and health outcomes	3% for both costs and health outcomes	3.5% for both costs and health outcomes
Perspective	Publicly funded health care system	Societal perspective	Societal perspective	Public payer perspective	Societal perspective	Perspective of the NHS and Personal Social Services (PSS)

* exchange rate on 4 July, 2013.

AAD: antiarrhythmic drug; AF: atrial fibrillation; CHADS2 acronym: Cardiac failure, Hypertension, Age ≥75 years, Diabetes, prior Stroke; CUA: Cost-Utility Analysis; HF: Heart Failure.

One of the models has a strange design. Reynolds et al.[11] mention to include patients refractory to one or more AADs. However, referring to their model, the authors state that “patients initially receive a first-line drug (sotalol or flecainide) and enter the “well 1st drug” state. In the event of toxicity or therapeutic failure, they proceed to treatment with amiodarone (“well amio” state), and in the event of amiodarone failure are treated with rate control (“RC/AC”).” It is important in modelling incremental costs and effects that both the intervention and control group start at the same, i.e. in this case after AAD failure.

Table S2: Information on costs (part 1: CA procedure and complications)

Reference	Assasi et al., 2010	Chan et al., 2006	Eckard et al., 2009	Ollendorf et al., 2010	Reynolds et al., 2010	Rodgers et al., 2008<br>McKenna et al., 2009
**AF ablation cost per procedure**	$9590	$16 500	$9860	$11 231 (without complications)	$15 000	£9810
Average number of procedures	1.27	1.30	1.40	Not reported in overview table	1.25	1.30
Procedural complications	Stroke: 0.3%<br>TIA: 0.2%<br>Cardiac tamponade: 0.8%<br>PV stenosis: 1.6%	Death: 0.1%<br>Stroke: 0.8%<br><br>Cardiac tamponade: 0.7%<br><br>Atrio-esophageal fistula: 0.2%<br>Other: 0.3%	Complications: 3% (Serious complications include: tamponade, bleeding, pulmonary vein stenosis, stroke and oesophageal fistulas)	Death: 0.1%<br>Stroke: 0.4%<br>Minor complications: 3.7%<br>Major complications: 1.3%	Procedural death: 0.05%<br>Stroke: 0.3%<br>TIA: 0.4%<br>Cardiac tamponade: 0.8%<br>PV stenosis: 0.4%<br>Vascular access: 1.2%<br>Pneumothorax / hemothorax: 0.18%<br>Phrenic nerve palsy: 0.1%	Operative death: 0.05%<br>Stroke: 0.28%<br><br>Cardiac tamponade: 1.22%<br>PV stenosis: 0.74%
Cost procedure complications	Tamponade: $5842<br>PV stenosis: $8487<br>stroke: $14 872<br>TIA: $4296	Complications from ablation: $11 000 (an average of complication costs from tamponade and stroke)<br>Atrioesophageal fistula: $50 000	$2190	AF ablation cost with complications: $17 024	Tamponade: $7500<br>PV stenosis: $7800<br>Stroke: $8200<br>TIA: $8200<br>Vascular access: $8000<br>Pneumothorax / hemothorax: $13 000	Tamponade: £815<br>PV stenosis: £3217

TIA: Transient ischemic attack; PV: Pulmonary vein.

Table S3: Information on costs (part 2: drugs)

Reference	Assasi et al., 2010	Chan et al., 2006	Eckard et al., 2009	Ollendorf et al., 2010	Reynolds et al., 2010	Rodgers et al., 2008<br>McKenna et al., 2009
Cost AAD	Total annual cost amiodarone: $433.	Annual care with amiodarone: $1200.	Annual cost: $1640 (This cost includes hospitalisation, AAD medication and consultation; hospitalization being the major cost driver for AAD)	Annual drug cost amiodarone: $434	Cost well on amiodarone: $3500	The base-case analysis assumed that amiodarone would be administered in an outpatient setting for all patients: £154. Amiodarone (200 mg daily): £32 per year.
Cost rate control	/	Annual cost rate control: $400 (combination of digoxin and atenolol).	/	Annual drug cost:<br>- digoxin: $263<br>- atenolol: $80	/	/
Cost anti-coagulation	- Proportion of patients taking warfarin in both treatment groups: 0.44.<br>- Annual cost warfarin treatment and monitoring: $463 (5 mg per day: $75.30, monitoring cost: $387.54).<br>- Based on treatment algorithms in the RCTs: assumed that AF ablation patients discontinue warfarin three months after their procedure, resulting in different bleeding risks between AF ablation patients and AAD-treated patients.	- Cost annual care aspirin: $13<br>- Cost annual care warfarin (including every 4-week monitoring): $600<br>- In all treatment arms, patients received antithrombotic or anticoagulant therapy. Patients at moderate risk of stroke received warfarin, whereas patients at low risk of stroke received either warfarin or aspirin. Patients with sinus rhythm restored continued warfarin therapy for six more months before transitioning to the use of aspirin.	Warfarin: $770<br>All AF patients with at least one risk factor for stroke (CHADS2) benefit from anticoagulation treatment to reduce thromboembolic events.	Annual drug cost:<br>- aspirin: $23<br>- warfarin: $440<br>Anticoagulation would continue as appropriate regardless of whether AF had recurred.	- Long-term anticoagulation practices and related costs and complications are equivalent between groups.<br>- Rate control/ anticoagulation: $2800/year.	- All patients receive anticoagulants and/or aspirin.<br>Warfarin (5 mg daily): £19 per year.<br>Aspirin (75 mg daily): £20 per year.<br>(summary table mentions the following use: Warfarin 64.0%, Aspirin 27.3%, and None 8.7%)

AAD: antiarrhythmic drug; AF: atrial fibrillation; CHADS2 acronym: Cardiac failure, Hypertension, Age ≥75 years, Diabetes, prior Stroke.

Table S4: Information on costs (part 3: stroke and other costs)

Reference	Assasi et al., 2010	Chan et al., 2006	Eckard et al., 2009	Ollendorf et al., 2010	Reynolds et al., 2010	Rodgers et al., 2008<br>McKenna et al., 2009
Cost stroke	1st year:<br>- ischemic stroke: $61 413<br>- hemorrhagic stroke: $58 159<br>subsequent years:<br>- ischemic stroke: $6801<br>- hemorrhagic stroke: $5843	Stroke: $8900	1st year: $19 180.<br>Subsequent years: $4380 per year.	Acute cost (hospital care):<br>- no disability: $7932<br>- mild disability: $10 075<br>- moderate/severe disability: $15 235<br>Annual cost:<br>- mild disability: $2990<br>- moderate/severe disability: $26 450	Stroke: $8200	1st year: £9431.<br>Subsequent years: £2488 per year.
Other costs	- Follow-up after CA: 1st year: $666 (three cardiologist consultations and CT scan). No follow-up costs after the first year.<br>- acute cost of pulmonary toxicity: $22 434.<br>- irreversible pulmonary toxicity: annual cost of $3799.<br>- Major gastrointestinal bleed: $6023.	Single event - hospitalization:<br>- Amiodarone pulmonary toxicity: $8600<br>- Intracranial bleed or stroke: No residual defects ($6400); Mild residual defects ($7830); Moderate to severe residual defects ($12 490)<br>- Extracranial haemorrhage: $3730<br>Annual care:<br>- Intracranial bleed or stroke: Mild disability ($2600); Moderate to severe disability ($23 000)<br>- Pulmonary toxicity caused by amiodarone: $3500		Drug toxicity:<br>- reversible: $100<br>- acute amiodarone pulmonary toxicity: $4250<br>- chronic amiodarone pulmonary toxicity: $4025<br>Haemorrhage & ICH:<br>- haemorrhage, not ICH: $3750<br>- ICH, no disability: $4295<br>- ICH, mild disability: $6048<br>- ICH, moderate/severe disability: $9536<br>Annual costs:<br>- mild disability: $2990<br>- moderate/severe disability: $26 450	- Well post ablation: 1st year: $1300, then $200/year.<br>- Cost well on 1st line drug: $4000<br>- Drug toxicity 1st line drug: Fatal: $10 000, Nonfatal: $5100.<br>- Amiodarone toxicity: Fatal: $10 000, Nonfatal: $5000.	- Costs of community and hospital-based care related to AF, including general practitioner consultations, anticoagulation visits and hospital costs. An annual amount of £646 was estimated for these costs.<br>- Toxic event: £1497.<br>- Reversale toxicity: £0.43 per day.<br>- Irreversible toxicity: £158 per year.<br>- Major bleed: £1573 per year.<br>- Minor bleed: £87 per year.

CA: catheter ablation; ICH: Intracranial haemorrhage.

Table S5: Risk information (part 1: stroke and bleeding risk)

Reference	Assasi et al., 2010	Chan et al., 2006	Eckard et al., 2009	Ollendorf et al., 2010	Reynolds et al., 2010	Rodgers et al., 2008<br>McKenna et al., 2009
Risk stroke	Annual probability of stroke by CHADS2 score:<br>0: 0.019<br>1: 0.028<br>2: 0.040<br>3: 0.059<br>4: 0.085<br>5: 0.125<br>6: 0.182<br>Stroke risk NSR = stroke risk AF x 0.625 (the inverse of 1.6).	Yearly stroke risk:<br>- In sinus rhythm: Moderate risk: 0.9%, Low risk: 0.5%<br>- Patients in AF: aspirin therapy: 2.3% and 1.1%, warfarin therapy: 1.3% and 0.7%, for moderate and low stroke risk, respectively.<br>- Stroke risk first month after AAD: 0.27%<br>- Stroke risk was adjusted linearly with a relative risk of 1.4 for each decade of age.	- AF: 1.5%<br>- free from AF: 1.5%	- Annual incidence rate: 0.019 (CHADS2 score = 0, vary by CHADS2 score)<br>- Reduced risk of stroke (secular trend): 0.315<br>- RRR stroke aspirin: 0.210<br>- RRR stroke warfarin: 0.680<br>- RR stroke after CA if NSR: 1.000 (0.15 in alternate scenario)	We assumed no benefit from ablation on stroke.	- Stroke risk for AF by CHADS2 score:<br>0: 1.9%<br>1: 2.8%<br>2: 4.0%<br>3: 5.9%<br>- Stroke risk for NSR: Hazard ratio for AF relative to NSR: 1.60.<br>- Stroke risk reduction with anticoagulation (RR): Warfarin vs placebo: 0.33, Warfarin vs aspirin: 0.59.
Bleed risk	without warfarin: 0.58%<br>with warfarin: 1.28%	- Warfarin therapy: 1.8% (age<75), 3.2 % (age ≥75)<br>'- Aspirin therapy: 1.2% (age<75), 1.5 % (age ≥75)<br>- Bleed outcome: Non-intracranial (85 %) and Intracranial (15 %) (of which Fatal (20%), Mild disability (67%), Moderate to severe disability (17%))<br>- Relative risk for recurrent bleeding: 1.5.		- rate of major haemorrhage: 0.006<br>- rate of major haemorrhage with aspirin: 0.012<br>- rate of major haemorrhage with warfarin: 0.018		- Annual probability bleed on warfarin: 2.40% (major), 15.80% (minor).<br>- Relative risk for bleeds comparing warfarin with aspirin: 0.58 (major), 0.45 (minor).<br>- Relative risk for bleeds comparing warfarin with no anticoagulant (OAC): 0.45 (major), 0.46 (minor).

AAD: antiarrhythmic drug; AF: atrial fibrillation; CA: catheter ablation; CHADS2 acronym: Cardiac failure, Hypertension, Age ≥75 years, Diabetes, prior Stroke; NSR: Normal sinus rhythm; RR(R): Relative Risk (Reduction).

Table S6: Risk information (part 2: toxicity and mortality risk)

Reference	Assasi et al., 2010	Chan et al., 2006	Eckard et al., 2009	Ollendorf et al., 2010	Reynolds et al., 2010	Rodgers et al., 2008<br>McKenna et al., 2009
Toxicity risk	- Annual probability of pulmonary toxicity while on AAD: 0.00832.<br>- The proportion of irreversible cases: 0.25.<br>- Probability of death after pulmonary toxicity: 0.091.	- Irreversible pulmonary toxicity: 0.5%<br>- Death from pulmonary toxicity: 0.1%<br>- Digitalis toxicity: 1.1%/year<br>- Beta blocker toxicity: 0.2%/year		Amiodarone toxicity:<br>- reversible toxicity: 0.104<br>- permanent disability from toxicity: 0.011<br>- fatal pulmonary toxicity: 0.000<br>Rate control toxicity (atenolol & digoxin):<br>- digitalis toxicity: 0.011<br>- beta blocker toxicity per year: 0.002	- Toxicity on IC AAD / sotalol: Fatal: 0.5% year one, then 0.32% per year; Nonfatal: 9.5% year one, then 1.28% per year.<br>- Toxicity on amiodarone: Fatal: 0.1% per year; Nonfatal: 9.9% year one, then 0.9% per year.	Side effects AADs:<br>- General toxicity: In year 1: 12.50%; In subsequent years: 6.25%.<br>- Withdrawal because of toxicity: In year 1: 10.00%, In subsequent years 5.00%.<br>- Probability of pulmonary complication given withdrawal: 15.19%<br>- Probability of irreversible pulmonary toxicity given withdrawal for pulmonary complication: 25.00%
(stroke) mortality	- Ischemic and hemorrhagic stroke mortality according to time (28 days, 1 year), age category and gender (see tables with full details in original text).<br>- For post-stroke mortality after one year, the general population mortality was increased by a factor of 2.3.	- Stroke outcome (Fatal, Moderate to severe disability, Mild disability, No disability): different for aspirin vs. warfarin.<br>- Relative risk for recurrent stroke: 2.<br>- Mortality was modified by a relative risk of 1.3 and 2.3 in patients without and with moderate-to-severe disability (from stroke or intra-cranial bleed), respectively.<br>- The model incorporated relative risk reductions of 17% and 33% in nonstroke vascular mortality by aspirin and warfarin, respectively.<br>- AAD: Relative risk for non-cardiovascular mortality: 1.08.	The ´post stroke´ health state implies an elevated mortality risk.	- Probability of death due to stroke: 0.179<br>- RR of death with mild disability: 1.3<br>- RR of death with moderate/severe disability: 2.3<br>(probability mild or moderate/severe disability with stroke: 0.411 or 0.300)<br>- RRR vascular death due to aspirin: 0.170<br>- RRR vascular death due to warfarin: 0.330<br>- Amiodarone: probability of death with cardioversion: 0.010	All patients face a background rate of mortality based on their age and sex.<br>Based on the low estimates of fatality from procedural complications or drug toxicity, projected all-cause mortality was equivalent between groups (7.7% ablation versus 7.8% AAD).	- Mortality risk from stroke (RR): 1st year: 7.40, subsequent years: 2.30.<br>- Probability of death given irreversible pulmonary toxicity: 20%.

AAD: antiarrhythmic drug; RR(R): Relative Risk (Reduction).

Table S7: Efficacy of intervention and comparator(s)

Reference	Assasi et al., 2010	Chan et al., 2006	Eckard et al., 2009	Ollendorf et al., 2010	Reynolds et al., 2010	Rodgers et al., 2008 McKenna et al., 2009
NSR						
CA	75.6% (Probability of AF ablation patients being in NSR at one year: 0.756 (2.93 x 0.258))	80% (Efficacy rate of 80%)	78% (Probability of AF free at 12 months: 0.780.)	Paroxysmal AF: 82.1% Persistent AF: 69.8%	90% (The model was calibrated to achieve a 10% overall failure rate with the ablation strategy. Recurrence after 1st ablation (6 months): 40%; AAD success post 1st ablation: 30%; Redo ablation 25%; Recurrence after 2nd ablation 50%; Success on drugs after 2nd ablation (6 months): 35%; Recurrence on IC AAD / sotalol (no ablation) (over 12 months): 75%.)	74 -84% (Probability of freedom from AF at 12 months: - Analysis 1 (RCT evidence): 0.8405 - Analysis 2 (RCT and case series evidence): 0.7404 - Analysis 3 (RCT and Cappato et al., 2005 evidence): 0.7867)
AAD	25.8% (Probability of AF ablation patients being in NSR at one year: 0.258)	85% (first line) (Overall cardioversion success: 85%)	9% (Probability of AF free at 12 months: 0.090)	83.3% (first line)	35% (Recurrence on amiodarone (no ablation) over 12 months: 65%)	24-37% (Probability of freedom from AF at 12 months: - Analysis 1: 0.3682 - Analysis 2: 0.2428 - Analysis 3: 0.3116)
rate control	/	38%	/	38%	/	/
AF recurrence						
CA	Annual probability of AF recurrence: 3.6%.	Annual relapse rate back to AF: 2%.	Risk ratio CA vs AAD: 0.1017.	Paroxysmal AF: 8.5% Persistent AF: 14.9%	See first row of this table	Annual rate of reversion to AF: 3.35%.
AAD	22.1%	30% in first 6 months, 5% yearly thereafter	Rate of AF in AAD: 2.4423.	9.7%	No data in overview table after 12 months	28.83%
rate control	/	Annual relapse rate: 5%	/	9.7%	/	/

AAD: antiarrhythmic drug; AF: atrial fibrillation; CA: catheter ablation; NSR: Normal sinus rhythm; RR(R): Relative Risk (Reduction).

Table S8: Utilities in the economic evaluations

Reference	Assasi et al., 2010	Chan et al., 2006	Eckard et al., 2009	Ollendorf et al., 2010	Reynolds et al., 2010	Rodgers et al., 2008<br>McKenna et al., 2009
Utilities	- NSR: age- and gender-specific general population utility values (going from 0.71 to 0.91, see details in original document).<br>- AF: disutility of 0.046.<br>- Stroke: 0.46 (post ischemic) and 0.28 (post hemorrhagic).<br>- CA complications: disutility of 1.0 for seven days.<br>- Pulmonary toxicity: disutility of 1.0 for duration of related hospitalization (mean 13 days).<br>- Irreversible pulmonary toxicity: utility weight of 0.6 in each cycle.	a) Permanent quality-of-life adjustment:<br>- Treatment strategy: Well in sinus rhythm (1.0), Aspirin (0.998), Warfarin (0.987), Amiodarone (0.987).<br>- Stroke or intracranial bleed: Mild residual defect (0.76), Moderate to severe residual defect (0.39)<br>- Persistent pulmonary toxicity (0.6)<br>b) Short-term disutilities for clinical events (stroke, hemorrhage, drug toxicity, and complications for ablation):<br>- Disutility value of 0.5 for the duration of the event.	- QALY-weights for males in normal population:<br>Age >69: 0.830<br>Age 70-79: 0.800<br>Age 80<: 0.740<br>- Decrement for AF: 0.100.<br>- Decrement for stroke: 0.250.	- Well in NSR (male, 60): 0.827 (varies by age & sex)<br>- AF: -0.065<br>- Comorbidities: HF (-0.0635), diabetes (-0.0351), hypertension (-0.0250), previous stroke or TIA (-0.0524), QoL (short-term) morbidity (0.5)<br>- procedure complications: -0.5<br>- cardioversion: -0.016 (3 days)<br>- CA: -0.004 (2.7 days, 4.7 days with minor complication), with major complication: -0.010<br>- permanent disability: -0.049<br>- amiodarone, aspirin, digoxin/atenolol: -0.002<br>- warfarin: -0.013<br>- acute drug toxicity: -0.4<br>- amiodarone pulmonary toxicity: -0.043 (3 days)<br>- ICH with mild or moderate/severe disability: -0.052 (10 days) or -0.305 (14 days)<br>- Stroke mild or moderate/severe disability: -0.052 (10 days) or -0.305 (14 days)	a) Chronic States<br>- Well after CA: 0.79<br>- Well on drugs: 0.79<br>- Rate control / anticoagulation: 0.725<br>- Post major stroke: 0.39<br>- Post minor stroke: 0.76<br>b) Disutility short-term events<br>- Nonfatal drug toxicity: 7 days<br>- Telemetry admission: 3 days<br>- Ablation complication: 4 days	- Reference point: utility of general population.<br>- Decrement for NSR: CA 0.0000, AADs 0.0199.<br>- Decrement for AF: CA 0.0034, AADs 0.0925.<br>- Stroke: Non-disabled stroke (year 1 and post year 1) 0.74, Disabled stroke (year 1 and post year 1) 0.38, Combined stroke (assuming 30.9% disabled) 0.63.<br>- Decrement pulmonary toxicity: 0.0329<br>- Decrement non-pulmonary toxicity (days of perfect health lost): 1 day.<br>- Decrement bleeding event (days of perfect health lost): 1 day.

AAD: antiarrhythmic drug; AF: atrial fibrillation; CA: catheter ablation; ICH: Intracranial haemorrhage; NSR: Normal sinus rhythm; QALY: Quality-Adjusted Life Year; QoL: Quality of Life; TIA: Transient ischemic attack.

Table S9: Results of the economic evaluations

Reference	Assasi et al., 2010	Chan et al., 2006	Eckard et al., 2009	Ollendorf et al., 2010	Reynolds et al., 2010	Rodgers et al., 2008<br>McKenna et al., 2009
Base case analyses	The primary economic analysis found the ICER of AF ablation compared to anti-arrhythmic medication to be $59 194 per quality-adjusted life year (QALY) in patients with a CHADS2 risk score of two, and for whom at least one anti-arrhythmic medication had failed.	In 65-year-old subjects with AF at moderate stroke risk, relative reduction in stroke risk with an 80% CA efficacy rate for sinus rhythm restoration would need to be ≧42% and ≧11% to yield ICERs <$50 000 and $100 000 per QALY, respectively.<br>Because amiodarone was both less effective and more costly, it was dominated by rate control therapy.	The RFA treatment strategy was associated with reduced costs ($25 460 vs $30 440) and an incremental gain in QALYs (9.46 QALYs vs 8.68 QALYs) compared to the AAD treatment strategy.	Secondary CA:<br>- 60, male, Paroxysmal AF: $37 808<br>- 65, male, CHF and Persistent AF: $73 947<br>- 75, male, DM HT Persistent AF: $96 846<br><br>Primary CA:<br>- 60, male, Paroxysmal AF: $22 172<br>- 65, male, CHF and Persistent AF: $60 804<br>- 75, male, DM HTN Persistent AF: $80 615	In the base case scenario, cumulative costs with the CA and AAD strategies were $26 584 and $19 898, respectively. Over 5 years, quality-adjusted life expectancy was 3.51 QALYs with CA versus 3.38 for the AAD group. The ICER for CA versus AAD was thus $51 431 per QALY.	There appears to be little variation across the different CHADS2 scores in terms of the ICER itself (ranging from £7763 to £7910 per additional QALY). At a threshold of £20 000 per QALY there is very little uncertainty surrounding the cost-effectiveness results. The probability that CA is cost-effective at this threshold varies from 0.981 to 0.992 across the separate risk groups.
Sensitivity	- No difference in utility between normal sinus rhythm and AF health states: $221 839/QALY.<br>- Restoring normal sinus rhythm has no impact on stroke: $86 129/QALY.<br>- 20-year time horizon: ablation becomes less costly and more effective than AAD.	- Higher and lower CA efficacy rates would require correspondingly lower and higher stroke risk reduction for equivalent ICER thresholds.<br>- In patients at low stroke risk, CA was unlikely to be cost-effective.	The results were sensitive to whether long-term quality of life improvement is maintained for the RFA treatment strategy and the risk of stroke in the different atrial fibrillation health states.	Secondary CA: 5 years<br>- 60, male, Paroxysmal AF: $193 272<br>- 65, male, CHF and Persistent AF: $267 261<br>- 75, male, DM HT Persistent AF: $294 599<br>Primary CA: 5 years<br>- 60, male, Paroxysmal AF: $105 907<br>- 65, male, CHF and Persistent AF: $161 090<br>- 75, male, DM HT Persistent AF: $171 729	- The model results were most sensitive to the time horizon, the cost of ablation, and to the relative utility weights of successful ablation versus unsuccessful drug therapy.<br>- Time horizon: 3 years: $157 000/QALY; 10 years: <$1000/QALY.<br>- Utility: ICER was $100 000/QALY with utility difference of 0.04 or greater. Larger differences resulted in quite favourable	Each of the different scenarios explored as part of the sensitivity analysis is then undertaken assuming a CHADS2 score of 1, considered to provide the most representative risk for this patient group:<br>- Results of the 5-year analysis show that the ICER for CA is within the range of conventional thresholds in the NHS: CHADS2 score 0:<br>£27 745/QALY; CHADS2

	- CHADS2: Score 0: $68 822/QALY; Score 4: $44 652/QALY.		QoL: if no decrement in QoL from AF: all strategies provide nearly identical total QALYs, pure rate control strategy is the most effective.	ICERs, whereas smaller differences yielded ICERs in the economically unattractive range.	score 3: £20 831/QALY. - No influence on stroke risk: lifetime analysis: £9237/QALY; 5-year analysis: £37 997/QALY.

AAD: antiarrhythmic drug; CA: catheter ablation; CHADS2 acronym: Cardiac failure, Hypertension, Age ≥75 years, Diabetes, prior Stroke; CHF: congestive heart failure; DM: Diabetes mellitus; HT: Hypertension; ICER: Incremental cost-effectiveness ratio; QALY: Quality-Adjusted Life Year; QoL; Quality of Life.

Table S10: Conclusions of the economic evaluations

Reference	Assasi et al., 2010	Chan et al., 2006	Eckard et al., 2009	Ollendorf et al., 2010	Reynolds et al., 2010	Rodgers et al., 2008<br>McKenna et al., 2009
Conclusion	The primary economic evaluation using a five-year time horizon found the incremental cost per QALY of AF ablation compared with AAD to be $59 194. These findings were similar to those of other published economic evaluations. The cost-effectiveness of AF ablation was found to be more favourable when longer time horizons were used.	In patients with AF, LACA is unlikely to be cost-effective in patients at low risk for stroke. In moderaterisk patients, LACA may be cost-effective if sufficiently high LACA efficacy rates in restoring sinus rhythm translate into lower morbidity. Our analyses may help in designing future clinical trials that compare ablation with medical therapy by providing estimates for LACA efficacy and stroke risk reduction needed in order to demonstrate both clinical efficacy and cost-effectiveness.	In conclusion, the RFA treatment strategy was associated with reduced cost and an incremental gain in QALYs and was considered a cost-effective treatment strategy compared to the AAD in a lifetime perspective, despite higher initial intervention costs.	No explicit conclusion on the cost-utility of CA.<br><br>Conclusion on the efficacy of CA:<br>a) 60, male, paroxysmal AF:<br>- secondary CA: high certainty of a small benefit.<br>- primary CA: unproven with potential.<br>b) 65, male, long-standing persistent AF and HF<br>- primary/secondary CA: unproven with potential.<br>c) 75, male, hypertension and diabetes mellitus and persistent AF<br>- primary/secondary CA: insufficient	RFA with/without AAD for symptomatic, drug-refractory paroxysmal AF appears to be reasonably cost-effective compared with AAD therapy alone from the perspective of the US health care system, based on improved quality of life and avoidance of future health care costs.	The overall conclusions regarding the cost-effectiveness of RFCA appear to require that the QoL benefits are maintained for more than 5 years and/or that NSR has prognostic value in preventing the risk of stroke. If neither of these is considered to be realistic then the cost-effectiveness of RFCA remains highly uncertain.

AAD: antiarrhythmic drug; AF: atrial fibrillation; CA: catheter ablation; LACA: Left atrial catheter ablation; NSR: Normal sinus rhythm; RF(C)A: Radiofrequency (catheter) ablation.
